# Holocephalan Embryo Provides New Information on the Evolution of the Glossopharyngeal Nerve, Metotic Fissure and Parachordal Plate in Gnathostomes

**DOI:** 10.1371/journal.pone.0066988

**Published:** 2013-06-17

**Authors:** Alan Pradel, Dominique Didier, Didier Casane, Paul Tafforeau, John Graham Maisey

**Affiliations:** 1 Department of Vertebrate Paleontology, American Museum of Natural History, New York, New York, United States of America; 2 Department of Biology, Millersville University, Millersville, Pennsylvania, United States of America; 3 Laboratoire Evolution, Génome et Spéciation, UPR 9034 CNRS, Gif-sur-Yvette, France, and Université Paris Diderot, Paris, France; 4 European Synchrotron Radiation Facility, Grenoble, France; Laboratoire de Biologie du Développement de Villefranche-sur-Mer, France

## Abstract

The phylogenetic relationships between the different groups of Paleozoic gnathostomes are still debated, mainly because of incomplete datasets on Paleozoic jawed vertebrate fossils and ontogeny of some modern taxa. This issue is illustrated by the condition of the glossopharyngeal nerve relative to the parachordal plate, the otic capsules and the metotic fissure in gnathostomes. Two main conditions are observed in elasmobranchs (shark and rays) and osteichthyans (bony fishes and tetrapods). The condition in the other chondrichthyan taxon, the holocephalans, is still poorly known, and without any information on this taxon, it remains difficult to polarize the condition in gnathostomes. Based on the anatomical study of an embryo of the holocephalan *Callorhinchus milii* by means of propagation X-Ray Synchrotron phase contrast microtomography using both holotomography and single distance phase retrieval process, we show that, contrary to what was previously inferred for holocephalans (i.e. an osteichthyan-like condition), the arrangement of the glossopharyngeal nerve relative to the surrounding structure in holocephalans is more similar to that of elasmobranchs. Furthermore, the holocephalan condition represents a combination of plesiomorphic characters for gnathostomes (e.g., the glossopharyngeal nerve leaves the braincase via the metotic fissure) and homoplastic characters. By contrast, the crown osteichthyans are probably derived in having the glossopharyngeal nerve that enters the saccular chamber and in having the glossopharyngeal foramen separated from the metotic fissure.

## Introduction

Chondrichthyans are a clade of cartilaginous fishes comprising two, morphologically distinct modern sister groups: the elasmobranchs (sharks, skates, and rays) and the holocephalans (ratfishes). The relationships between these two monophyletic groups and the other gnathostomes (jawed vertebrates) are easy to establish when only extant forms are considered: both morphological and molecular data support the monophyly of osteichthyans and chondrichthyans, together forming the modern gnathostomes, sister group of the cyclostomes (hagfishes and lampreys) [1]–[3]. The phylogenetic relationships between the different groups of gnathostomes are much more debated when Paleozoic forms are considered. Debate about the phylogenetic position of particular gnathostome taxa is a result of incomplete datasets on Paleozoic fossils and ontogeny of some modern gnathostomes. Conflicting phylogenetic hypotheses results in conflicting diagnoses of taxa either stem- or crown- taxa by unambiguous synapomorphies.

This issue is illustrated by the condition of the glossopharyngeal nerve relative to the parachordal plate, the otic capsules and the metotic fissure in gnathostomes.

A ventral metotic fissure is present in all gnathostome embryos. It separates the parachordal plate from the otic capsule at early ontogenetic stages. This fissure generally closes during ontogeny by the formation and closure of anterior and posterior basicapsular commissures between the parachordal plate and the otic capsule [4]. The two commissures form the anterior and posterior boundaries of the embryonic basicapsular fenestra, which corresponds to the vestibular fontanelle in adult gnathostomes [5]. The glossopharyngeal nerve leaves the embryonic neurocranium through the basicapsular fenestra in gnathostome embryos [4]. However, the subsequent ontogeny of the glossopharyngeal nerve follows different trajectories in modern adult osteichthyans and elasmobranchs. In osteichthyans, the medial wall of the otic capsule is unchondrified and the glossopharyngeal nerve leaves the cranial cavity via a foramen located on the lateral wall of the saccular chamber of the otic capsule [4]. Furthermore, there is no hypotic lamina (i.e. a lateral extension of the parachordal plate which floors the glossopharyngeal canal), and the glossopharyngeal foramen is separated from the metotic fissure (or a remnant of the fissure, such as the vestibular fontanelle). By contrast, in modern elasmobranchs, the medial capsular wall is chondrified, the glossopharyngeal nerve leaves the braincase via the remnant of the metotic fissure, and the nerve is floored by the hypotic lamina, so that it has a more horizontal course than in osteichthyans after leaving the endocranial cavity. The course of the glossopharyngeal nerve relative to the otic capsule in elasmobranchs has been extensively discussed: De Beer [4] and Jollie [6] described the glossopharyngeal nerve as passing external to the otic capsule, whereas Holmgren [7], [8] argued that the nerve actually entered the auditory capsule. Unfortunately, no subsequent detailed studies have been presented, although the interpretation of De Beer [4] and Jollie [6] is commonly accepted.

The arrangement of the glossopharyngeal nerve in modern holocephalans is more problematic, and remains unclear. Earlier studies on chondrichthyan ontogeny were mostly focused on the elasmobranchs and little was done on the ontogeny of extant chimaeroids [4], [7], [8]. Furthermore, the few available studies on the development of chimaeroids are incomplete. In the past, it has been assumed that the chondrocranial condition in chimaeroids was the same as in osteichthyans, because, in both taxa, the medial capsular wall is unchondrified, a hypotic lamina is absent, and the glossopharyngeal nerve foramen is located farther ventrally on the braincase than in elasmobranchs [8]. However, De Beer [4] described the otic capsule of the holocephalans as possessing a complete floor, with the glossopharyngeal nerve remaining external to it.

In order to clarify the evolution of these features in gnathostomes, it is necessary to perform new anatomical studies.

Extant holocephalans, especially *Callorhinchus milii*, the sister genus of all other extant chimaeroids, is receiving more and more interest, since its mitochondrial genome was sequenced, showing that this species is a very useful model for evolutionary genomic studies on gnathostomes [9]. This species is also used in recent evo-devo studies because it displays some key anatomical characteristics for the understanding of the evolution of early gnathostomes [10]. Recently, paleontological studies using X-ray synchrotron (SRμCT) holotomography have provided some new information about the early evolutionary history of the holocephalans [11]. Nevertheless, paleontological and evo-devo studies are constrained by the paucity of developmental data on extant chimaeroids. New data on the embryological development and on the detail anatomy of holocephalans are thus essential for the understanding of the evolution of jawed vertebrates.

Here we provide for the first time a detailed description of the condition of the glossopharyngeal nerve in relation to its surrounding structures (otic capsules, parachordal plate and metotic fissure) in a late embryo of an extant chimaeroid, *Callorhinchus milii*. This anatomical study is performed using propagation phase contrast SRμCT protocole (PPC-SRµCT) recently applied to vertebrate embryos ([12] and the present work). SR-μCT allows the observation of both mineralized (e.g., skull and teeth) and soft (e.g., nervous system) tissues, *in situ*, with a high resolution and without any artifact due to manual dissection. We also provide here a 3D reconstruction of the head and otic capsules of the dogfish *Scyliorhinus canicula*, as well as a discussion about the comparative embryology and evolution of these structures, defining synapomorphies for the glossopharyngeal nerve and associated structures in the crown gnathostome taxa.

## Materials and Methods

The *Scyliorhinus canicula* specimen was treated according to the French and European regulations for handling of animals in research. It is a juvenile specimen, which had reached the stage of independent feeding at the time of killing. It was grown in the Laboratoire Evolution, Génome et Spéciation, UPR 9034 CNRS, Gif-sur-Yvette, France, which has appropriate licence to breed such a species provided by the “direction départementale des services vétérinaires” of the French Republic (number 2009-DDSV-031, April 15^th^ 2009). The embryo used in this study is an aquatic, non-endangered, non-mammalian vertebrate and therefore did not require special authorizations. All efforts were made to minimize suffering.

The specimen was anaesthetized with MS222 (Sigma) before the sacrifice. It was fixed with 4% paraformaldehyde and preserved in 100% methanol solution. It was scanned in 2008 using holotomographic approach similar to the one described in [11]. The voxel size was 7.45 microns, and 4 propagation distances (20, 150, 400 and 900 mm) were used in order to retrieve the phase maps. We used a beam at 20 keV monochromatized using a double Bragg Si 111 crystal monochromator. For each distance, 1200 projections of 0.6s each over 180 degrees were used. The specimen was in a cylindrical container filled with 100% ethanol solution. By replacing the water in tissues, 100% ethanol strongly increases the differential contrast of the tissues. When coupled with phase contrast X-ray imaging, this permits the collection of very high quality 3D data on soft tissues, up to the cellular level.

The embryo of *Callorhinchus milii* was collected from a spawning site in the Marlborough Sounds, New Zealand. The appropriate protocols for animal welfare were followed according to guidelines published by the American Society of Ichthyologists and Herpetologists.

The egg capsule was collected by SCUBA diving. The egg capsule was opened within hours of collection and the embryo was removed and dissected from the yolk sac. Embryos were preserved in 10% formalin in seawater. After a minimum of one month in fixative, embryos were washed and transferred to 70% ethanol for long-term storage (see [13] for more information). Permits to collect and export the embryos were obtained from the New Zealand Ministry of Fisheries (now NIWA) and permission and support of research activities in the Marlborough Sounds was granted by the New Zealand Department of Conservation. Copies of the permits are held by the Academy of Natural Sciences in Philadelphia where the specimen was deposited.

The specimen studied here is at embryonic stage 36 [14]. It was scanned on the ID19 beamline of the ESRF using an isotropic voxel size of 30 microns, with a single propagation distance of 4 m. The specimen was scanned in a 75% ethanol solution. The beam was produced by the U17.6 undulator closed at a gap of 13.5 mm, filtered by 2 mm of aluminum and 1mm of copper. Such a configuration allows isolating mostly a single harmonic from the original insertion device spectrum, with an effective energy of 55 keV. In order to obtain differential contrasts for the different tissues of the specimen, a single distance phase retrieval process was used [15], [16]. This very simple approach allows generating 3D volumes with high sensitivity, contrast and signal to noise ratio, while retaining high resolution and avoiding the propagation fringes typical of edge detection scans. The results are very close to what can be reached using the much more complex holotomographic approach, for a scanning time typically 10 times shorter, and a straightforward and rapid processing.

The full sample was scanned in 6 sub-scans of 4 minutes each, using 2499 projections of 0.1 s each over 360 degrees. The detector was based on a 750 µm thick LuAG scintillator, coupled to a FReLoN (Fast Readout Low Noise) CCD camera through a lens based optic system.

Volumes were reconstructed using ESRF software PyHST, that includes the single distance phase retrieval algorithm [15]. Sub-volumes were corrected for ring artefacts, and then concatenated to generate a single stack of 16bits tif slices.

Segmentation and 3D rendering were performed with MIMICS® 15.01 64 bit software (Materialise Inc. NV, Leuven, Belgium).

## Results

**Figure 1:**
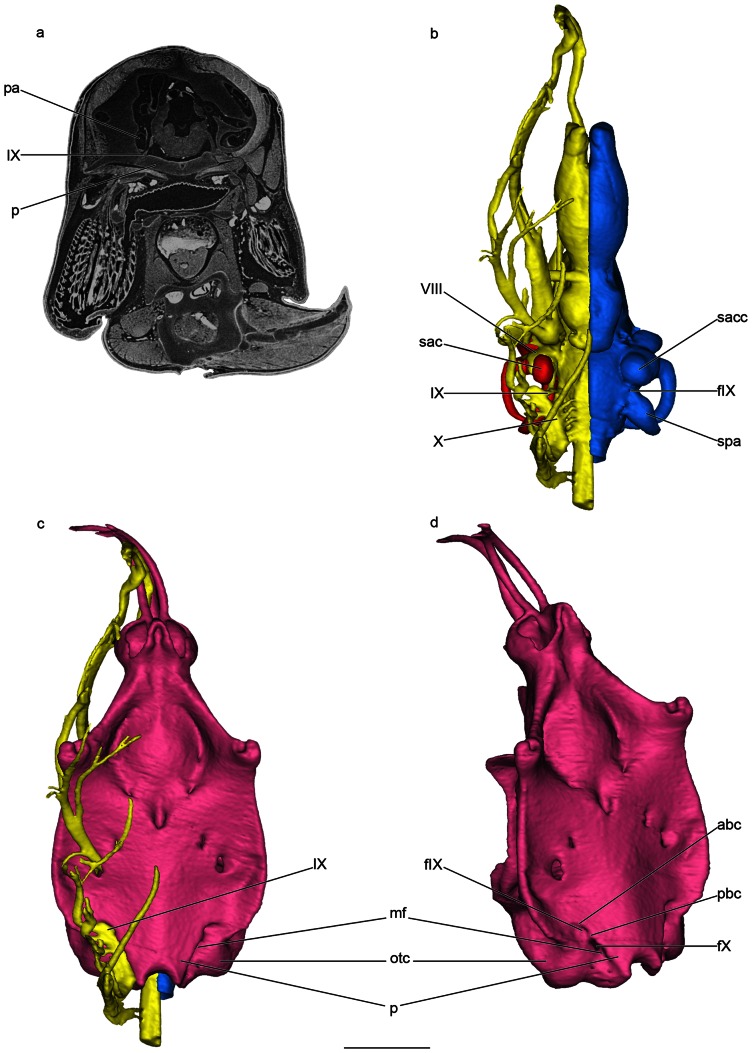
Microtomographic slice and surface renderings generated from PPC-SRµCT of a late embryo of *Callorhinchus milii*. a, transverse microtomographic slice through the posterior end of the otic capsule. b, ventral view of the brain, the otic capsule and the endocranial cavity. c, ventral view of the braincase and cranial nerves. D, oblique ventrolateral view of the braincase, right side. Scale bar = 0.5 cm. Colors: pink, braincase; blue, internal cast of the endocranial cavity; red, membranous labyrinth of the otic capsule; yellow, brain and cranial nerves. Abbrevations: abc, anterior basicapsular commissure; fIX, foramen for the glossopharyngeal nerve; fX, foramen for the vagal nerve; mf, metotic fissure; otc, otic capsule; p, parachordal plate; pa, posterior ampulla of the membranous labyrinth; pbc, posterior basicapsular commissure; sac, sacculus of the membranous labyrinth; sacc, saccular chamber of the skeletal labyrinth; spa, posterior ampulla of the skeletal labyrinth; VIII, acoustic nerve; IX, glossopharyngeal nerve, X, vagal nerve.

The glossopharyngeal nerve of *Callorhinchus milii* (IX, Fig. 1a, b, c) originates from the ventrolateral surface of the anterior portion of the medulla (myelencephalon), posterior to the root of the auditory nerve (VIII, Fig. 1b), and anterior to the rootlets of the vagal nerve (X, Fig. 1b). Then, it runs over a short distance ventrolaterally to join the extracranial ganglion of the vagal nerve. After that point, it extends anteroventrally toward the first gill pouch and divides into three main branches: pretrematic, pharyngeal and posttrematic.

The glossopharyngeal nerve passes posterior to the chondrified base of the membranous medial capsular wall, behind the sacculus and ventral to the posterior ampulla (IX, sac, pa, Fig. 1a, b). Consequently, it does not pass through the membranous medial capsular wall, and remains separated from the membranous labyrinth throughout its length. The glossopharyngeal nerve leaves the endocranial cavity posterior to the saccular chamber and ventral to the posterior ampulla of the skeletal labyrinth (fIX, sacc, spa, fig. 1b). The nerve never actually enters the saccular chamber, and it exits the endocranial cavity ventral to the skeletal labyrinth. The glossopharyngeal nerve finally leaves the braincase ventrally, via a foramen located ventral to the otic capsule and lateral to the parachordal plate (fIX, otc, p, Fig. 1c, d).

In adult holocephalans the metotic fissure is closed. In late late embryo of *Callorhinchus* examined here, the embryonic metotic fissure is still evident on the ventral surface of the basicranium, between the parachordal plate and the otic capsules (mf, p, otc, fig. 1c, d), although it has already started to close with the formation of anterior and posterior basicapsular commissures (abc, pbc, Fig. 1d). The position of these two commissures determines the location of the glossopharyngeal nerve foramen (fIX, Fig. 1d), while the vagal nerve foramen (fX, Fig. 1d) is bounded by the posterior basicapsular commissure anteriorly. Although the basicranium of *Callorhinchus* is broad and flat, the parachordal plate does not flare laterally to form a hypotic lamina below the otic capsules (p, otc, fig. 1c, d).

**Figure 2:**
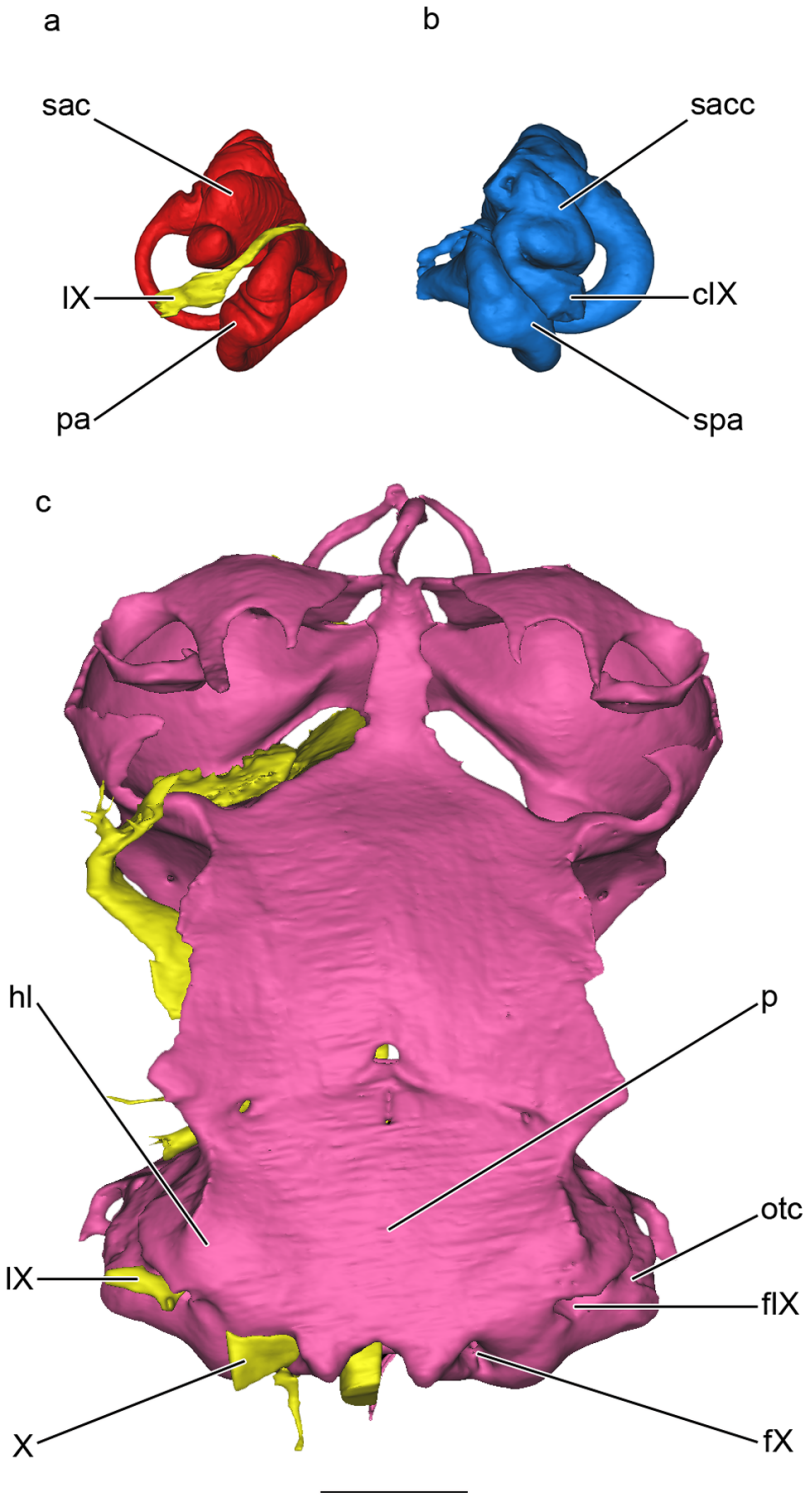
Surface renderings of *Scyliorhinus canicula* generated from SRµCT holotomography. a, ventral view of the right membranous labyrinth and glossopharyngeal nerve. b,.ventral view of the cast of the cavity of the left otic capsule. c, ventral view of the braincase and cranial nerves. Scale bar = 0.25 cm. Same colors and abbreviations as in figure 1. Additional abbreviation: hl, hypotic lamina.

In modern elasmobranchs, represented here by the dogfish *Scyliorhinus canicula*, the medial capsular wall is chondrified. The glossopharyngeal nerve passes through the base of this wall and then runs posteriorly, ventral to both the membranous and skeletal labyrinth of the otic capsule (IX, sac, pa, Fig. 2a; cIX, sacc, spa, Fig. 2b). The nerve finally leaves the braincase posteriorly through a foramen that is located ventral to the otic capsule (fIX, otc, Fig. 2c). The metotic fissure is closed during the ontogeny, leaving two foramina for the glossopharyngeal and vagal nerves (IX, X, fIX, fX, Fig. 2c). Contrary to *Callorhinchus*, the parachordal plate flares laterally to form a hypotic lamina, which is ventral to the otic capsule and forms the floor of the glossopharyngeal and vagal canals (p, hl, otc, IX, X, Fig. 2c).

## Discussion

The evolution of the characters described in the present paper is discussed in the light of the two main recently published phylogenetic assumptions [17], [18], which differ mainly in the position of some Paleozoic chondrichthyans (*Cobelodus* and *Akmonistion* are stem holocephalans and *Orthacanthus* is a stem elasmobranch in [17], whereas these are all resolved as stem chondrichthyans in [18]).

### The exit of the glossopharyngeal nerve relative to the metotic fissure

In the light of the first phylogenetic hypothesis [17], the metotic fissure remains open in adults of both some stem holocephalans (e.g., *Cobelodus* [19], *Akmonistion* [20]) and elasmobranchs (e.g., *Orthacanthus* [21]). The fissure is closed in other both stem elasmobranchs (e.g., the hybodont *Egertonodus* [22]) and holocephalans (e.g., *Iniopera* [23]), and in all modern chondrichthyans (e.g., the neoselachians *Notorynchus* [24] and *Scyliorhinus*, see above; the holocephalan *Callorhinchus*, see above). This implies that the closure of the metotic fissure in crown holocephalans and elasmobranchs is convergent.

Alternatively, in the light of the second phylogenetic hypothesis [18], the metotic fissure remains open in adults of stem chondrichthyans (e.g., *Pucapampella* [25]; *Doliodus* [26]; *Cobelodus* [19], *Akmonistion* [20]; *Cladodoides* [27], *Orthacanthus* [21]), whereas it is closed in adult crown chondrichthyans (e.g., the hybodont *Egertonodus* [22]; the neoselachian *Scyliorhinus,* see above; the stem holocephalan *Iniopera* [23]; the extant *Callorhinchus*, see above). Consequently, an open metotic fissure in adults appears to be the plesiomorphic condition for the total-group chondrichthyans, whereas a closed fissure is a derived character for crown chondrichthyans.

Besides the chondrichthyans, a persistent metotic fissure has been documented in stem osteichthyans (*Acanthodes* [28]; *Ligulalepis* [29]), stem actinopterygians (e.g., the paleoniscids *Mimia* and *Kansasiella* [30], [31]), stem dipnoans [32] and stem tetrapodomorphs [33]. An open metotic fissure is thus plesiomorphic for osteichthyans, as well as for crown gnathostomes. However, there is no evidence of a persistent metotic fissure in placoderms and the two jawless stem gnathostomes (“ostracoderms”) whose braincase is known, i.e. osteostracans and galeaspids, so that a closed fissure in adults appears to be plesiomorphic for the total-group gnathostomes.

In stem chondrichthyans, the glossopharyngeal and vagal nerves leave the braincase through the open metotic fissure [34]. In modern chondrichthyans, the fissure closes during ontogeny, except for the glossopharyngeal and vagal foramina. Consequently, a glossopharyngeal nerve passing through the metotic fissure (whether the latter is opened or closed) is a widespread feature of chondrichthyans. The same arrangement was recently revealed in the stem osteichthyan *Acanthodes* [28], suggesting that a glossopharyngeal nerve passing through the metotic fissure is plesiomorphic for crown gnathostomes. In crown osteichthyans, the glossopharyngeal nerve does not exit the braincase through the fissure; instead its foramen is located above and/or anterior to the vestibular fontanelle, which represents the anteriormost part of the embryonic metotic fissure plus the embryonic basicapsular commissure in adults [4], [5], [30], [31]. This osteichthyan condition may thus be considered as a derived feature among crown gnathostomes. Separation of the glossopharyngeal foramen from the basicapsular fenestra appears early in the ontogeny of extant osteichthyan braincases and seems to be related to the developement of the posterior basicapsular commissure (e.g., *Polypterus* [4]).

### The medial capsular wall and the course of the glossopharyngeal nerve relative to the otic capsule

In extant elasmobranchs and in *Callorhinchus*, the glossopharyngeal nerve does not leave the braincase cavity through the lateral wall of the saccular chamber (see above). In the iniopterygian (stem holocephalan) *Iniopera*, the glossopharyngeal nerve pierces the endocranial cavity at the base of the posteroventral corner of the saccular chamber ([23]: Figs. 26B, 28) and then leaves the braincase via a foramen located ventral to the otic capsule. The iniopterygian arrangement is more similar to that of *Callorhinchus* than that of osteichthyans, in which the glossopharyngeal canal is located more dorsally (more or less at the level of the middle of the saccular chamber, the posteroventral corner of which is confluent with the metotic fissure; e.g., *Kansasiella* [31]: Fig. 27). In stem chondrichthyans (e.g., *Cladodoides* [27]), as well as in *Acanthodes* [28], there is no evidence of a glossopharyngeal foramen on the lateral wall of the otic capsule, although a glossopharyngeal canal may have been present at the base of the posteroventral corner of the saccular chamber of the symmoriiform *Kawichthys* [18], as in *Iniopera*. In all likelihood, the glossopharyngeal nerve did not enter the otic capsule in *Acanthodes* and stem chondrichthyans, which may represent the plesiomorphic condition for crown gnathostomes. The condition in osteichthyans thus represents a derived condition for crown gnathostomes.

The glossopharyngeal nerve apparently entered the otic cavity in arthrodires [35], [36], but not in more inclusive placoderms (e.g., *Brindabellaspis*, *Macropetalichthys* [37]). This suggests that the glossopharyngeal nerve was primitively located beneath the otic capsule in gnathostomes, and that its passage through the otic capsule is a derived condition that was acquired independently by arthrodires and osteichthyans.

### The floor of the glossopharyngeal canal and the hypotic lamina

The hypotic lamina is a lateral expansion of the parachordal plate beneath the otic capsule in all extant elasmobranchs, forming the floor of the glossopharyngeal canal [21], [24]. A hypotic lamina is also documented in stem elasmobranchs (e.g., the hybodont *Egertonodus* [22]). When a hypotic lamina is present, the course of the glossopharyngeal nerve is horizontal and the nerve leaves the braincase either posteriorly (e.g., *Egertonodus*), or posterolaterally (extant elasmobranchs). Although the otic region is broad ventrally in extant chimaeroids, there is no evidence of a hypotic lamina (see above). In some stem holocephalans, such as *Iniopera*, the presumed parachordal region is keel-shaped, with sloping lateral margins, and the glossopharyngeal nerve leaves the braincase ventrally [23], as in *Callorhinchus*. According to [17]'s phylogenetic assumption, a hypotic lamina is however present in some other stem holocephalans (e.g., *Cobelodus* [19] and *Akmonistion* [20]), and in the stem chondrichthyans (e.g., *Cladodoides* [27]; *Doliodus* [26]).

Stem and crown osteichthyans do not possess a hypotic lamina [28]. Consequently, a lateral expansion of the parachordal encompassing the passage of the glossopharyngeal nerve within a canal or persistant metotic fissure may be a synapomorphy of chondrichthyans, but secondarily lost in some holocephalans. Alternatively, in the light of [18]'s phylogenetic assumption, in which *Cobelodus* and *Akmonistion* are resolved as stem chondrichthyans, the hypotic lamina is secondarily lost in total group holocephalans.

The presence or absence of a hypotic lamina may be related to the position of the branchial skeleton relative to the braincase. In elasmobranchs and many stem chondrichthyans, the branchial skeleton is located mostly behind the braincase, whereas in osteichthyans and holocephalans, it lies mostly beneath the neurocranium. Consequently, the glossopharyngeal nerve, which innervates the first branchial arch, has to exit the braincase posteriorly in elasmobranchs and stem chondrichthyans, and more anteroventrally in osteichthyans and holocephalans. The peculiar branchial skeleton (e.g., presence of a pharyngohyal element) and its position below the braincase in holocephalans are probably derived for chondrichthyans [13], [38], and this may provide a functional explanation for the absence of the hypotic lamina in chimaeroids.

## Conclusions

We have provided here new observations on the anatomy of a late embryo of an extant holocephalan by means of phase contrast Synchrotron X-Ray microtomography with single distance phase retrieval process. This information allows us to discuss the phylogenetic status of characters related to the glossopharyngeal nerve, otic capsule and parachordal plate in gnathostomes.

It is concluded that the morphology of the otic region in modern chimaeroids represents a combination of plesiomorphic characters (e.g., the glossopharyngeal nerve leaves the braincase via the metotic fissure) and homoplastic characters (e.g., the absence of an elasmobranch-like hypotic lamina, which is present in stem chondrichthyans but not in osteichthyans). By contrast, crown osteichthyans are probably derived in having the glossopharyngeal nerve entering the saccular chamber and in having the glossopharyngeal foramen separated from the metotic fissure (or vestibular fontanelle).

This work confirms the rapidly growing potential of X-ray synchrotron imaging techniques in biology and embryology. It vindicates Synchrotron radiation as a powerful tool for non-destructive in-situ imaging of both hard and soft tissues in living organisms.
